# Supplementary material for: Are carbon emissions trading and green financial instruments synergistic? -Comprehensive quantitative research based on content analysis
Source: PLoS One. 2024 Mar 7;19(3):e0298601. doi: 10.1371/journal.pone.0298601 (PMC10919601; doi:10.1371/journal.pone.0298601)
Supplement: S1 Data — (ZIP) [file pone.0298601.s001.zip › Data/地级市Z_F工作报告.zip!/╔╜╢1⁄2/╫═▓⌐╩╨/└┤╫╘╣1⁄2╓┌║┼í╛┬φ┐╦╩2╛▌═°í┐_2018.docx]

各位代表：

现在，我代表市人民政府，向大会作政府工作报告，请予审议，并请各位政协委员和列席会议的同志提出意见。

一、2017年政府工作回顾

刚刚过去的2017年，在中共淄博市委的坚强领导下，全市上下深入学习贯彻党的十八大、十八届历次全会和党的十九大精神，按照省第十一次党代会、市第十二次党代会和市十五届人大一次会议部署要求，团结奋进，创新实干，积极作为，全市经济社会实现平稳健康发展。地区生产总值达到4781.3亿元，同比增长7.4%；一般公共预算收入完成361.58亿元，同比增长6.1%；城镇居民和农村居民人均可支配收入分别达到39410元和16953元，同比均增长8.2%。我市成功入选全国首批老工业城市和资源型城市产业转型升级示范区，荣获全国文明城市“三连冠”，全国社会治安综合治理优秀市“三连冠”并获得“长安杯”。

（一）产业转型升级步伐加快。研究出台推动新旧动能转换的一揽子政策措施，年度市重大项目完成投资924亿元，战略性新兴产业项目占到工业项目数量的71%。传统产业转型升级明显提速,工业技改投资完成1260亿元，压减建陶产能5.8亿平方米，关停化工企业242家,电子商务、两化融合、“互联网+”进一步普及，智慧车间、个性化定制等新模式、新业态加快兴起。“四大招引工程”取得积极进展，与驻地央企合作实现新突破，东岳集团燃料电池膜、山东理工大学无氯氟聚氨酯化学发泡剂等自主研发技术取得重大成果。省级以上重点实验室、企业技术中心等创新平台达到317家，院士工作站和博士后科研平台分别达到87家和48家，张店区入选国家级双创示范基地，全市高新技术产业产值占比提高1.2个百分点。改革完善省级经济开发区体制机制，园区承载力和发展活力明显提升，产业集约集聚发展态势更加清晰。传化金泰公路港等一批现代服务业引领项目扎实推进，服务业占比提高1.2个百分点。农业转型升级十大行动深入实施，粮食生产实现“十五连丰”，确保了粮食安全。

（二）生态环境质量持续改善。以中央和部、省环保督察巡查为契机，坚持全域治理、精准治理、铁腕治理，扎实开展环境突出问题和秋冬季大气污染综合治理攻坚行动，做实覆盖镇（街道）、村（社区）的网格化监管机制，关停傅山焦化、宏源焦化、阳煤一化等一批高耗煤、高排放大户，取缔“散乱污”企业9523家，完成绿动力提升工程和11.3万户气代煤、电代煤年度改造任务，全市燃煤消耗总量下降到2990万吨以内，SO2、NO2、PM10、PM2.5平均浓度同比分别改善33.3%、13%、11.2%和14.9%。高标准推进重点流域综合整治，积极推行河长制，实施城镇污水处理厂提标改造，主要河流断面COD和氨氮平均浓度同比分别改善7.3%和34.8%。全面完成“1256”生态修复绿化工程，文昌湖、马踏湖、孝妇河湿地公园等一批高品质生态项目基本建成。以前所未有的力度压紧环保工作责任、严惩环境违法行为，凝聚形成了加强生态环保、推动绿色发展的强大思想共识和行动自觉。

（三）文化名城建设扎实推进。在顶层设计、项目建设、宣传推介、功能配套等方面同步发力，文化名城建设规划体系基本形成，齐文化传承创新示范区核心区建设进展顺利。周村古商城等61个文旅融合发展重点项目完成投资162亿元，国家全域旅游示范市创建加快推进。文化产业增加值占比达到4.64%，跻身第一批国家文化消费试点城市。城乡公共文化服务体系进一步完善，打造了“淄川文化云”、高青县“小微文化”、沂源县乡村文化理事会等一批文化惠民品牌。广泛开展群众性精神文明创建活动，全国农村精神文明建设工作经验交流会和全国文明校园创建工作推进会在我市召开。

（四）城市现代化功能加快提升。城市总体规划等重大规划编制进展顺利，土地利用总体规划调整取得重要成果，我市被列入全国“城市双修”试点和老旧小区改造试点。125项主城区及全市统筹城建重点项目完成投资183亿元，淄博新区、淄博经开区、文昌湖区、高铁新城等重点区域承载功能不断增强，济青高铁、济青高速、滨莱高速、S102济青线淄博段新改建等重点交通工程，以及人民路改造等30个市政路网项目顺利实施。全面完成“三改三建”年度任务，住宅小区物业管理实现全覆盖。全市公交集约化改革深入推进，城市网约车、共享单车快速发展。加强城市精细化管理，深入开展市容市貌和违法建设专项治理，拆除违法建设1290万平方米。积极推进美丽乡村标准化建设，完成608个村庄硬化“户户通”工程，提前一年半完成省定农村旱厕改造任务。

（五）改革开放向纵深推进。深化“三最”城市建设，取消行政权力事项52项、无谓证明和繁琐手续170项，便民服务中心实现镇（街道）全覆盖；积极推行“多证合一”和电子营业执照，全市新登记各类市场主体8.23万户，同比增长27.8%。新增上市企业2家、新三板挂牌企业9家、齐鲁股交中心挂牌企业104家，全国首家多层次资本市场资源联合培育基地在齐鲁股交中心设立。公共资源交易市、县（区）一体化平台加快建设，农村集体产权制度、不动产登记制度、投融资体制以及国有企业、教育、医疗等重点领域改革不断深化，全市新增国家级改革试点47项、省级改革试点102项，打造了以信息化推进改革、“六个一”督察机制等一批全面深化改革的“淄博实践”品牌。加大对外开放力度，实际使用外资同比增长14.7%，“淄博号”中欧国际货运班列开通运行，货物贸易进出口总额历史性突破100亿美元，同比增长29.6%。齐文化节、陶博会、新材料技术论坛等品牌展会影响力进一步提升。

（六）民生社会建设切实加强。完善稳定脱贫长效机制，实现农村低保线与扶贫线“两线合一”，市定现行标准下贫困人口基本实现“两不愁、三保障”。全市城镇登记失业率2.9%，参加各类社保人员突破1000万人次。新改扩建幼儿园107所、中小学校62所，“大班额”问题得到切实缓解，提前一年完成“全面改薄”任务。加快建设“健康淄博”，医疗重点项目、基层卫生服务机构标准化建设进展顺利，博山区入选全国智慧健康养老示范基地。省级食品安全先进市创建成效显著。参加全运会取得优异成绩。深化平安淄博、法治淄博建设，全力抓好安全生产、社会治安综合治理和金融风险防控，圆满完成十九大安保维稳任务，社会大局保持和谐稳定。统计、物价、广播电视、民族宗教、外事侨务、对台、防震减灾、人防、气象、史志、档案、住房公积金、关心下一代、妇女儿童、青少年、老龄、慈善、残疾人、红十字、对口支援等各项事业都取得了新成绩。

一年来，我们高度重视政府自身建设，自觉用习近平新时代中国特色社会主义思想武装头脑、指导实践、推动工作。牢固树立“四个意识”，扎实推进“两学一做”学习教育常态化制度化，坚定不移贯彻落实中央和省、市委决策部署。认真执行市人大及其常委会决议、决定，自觉接受人大、政协和社会监督，主动听取各民主党派、工商联和社会各界意见，认真办理人大代表建议、政协委员提案。坚持依法行政、从严治政，规范完善行政决策执行监督机制，驰而不息纠治“四风”，着力破解“为官不为”“落实不力”突出问题，政府执行力、公信力不断提升。

各位代表！过去的一年，全市经济社会发展迈上了新台阶、实现了新突破。这是省委、省政府和市委坚强领导的结果，是市人大、市政协和社会各界监督支持的结果，是全市广大干部群众团结一致、攻坚克难、共同奋斗的结果。在此，我代表市人民政府，向全市广大干部群众，向各民主党派、工商联、各人民团体和社会各界人士，向中央、省驻淄单位、驻淄部队和武警官兵、政法干警，向海内外所有关心支持淄博改革发展的同志们、朋友们，表示衷心的感谢并致以诚挚的敬意!

同时，我们也清醒认识到，我市经济社会发展还存在不少矛盾和制约，发展不平衡不充分的问题依然比较突出。主要是：产业转型升级任重道远。传统产业、重化产业占比较大，新兴产业尚未形成主导优势，科技创新对经济增长的贡献率不够高，投资特别是民间投资还不够活跃，具有重大支撑带动作用的产业项目偏少，经济发展质效需要切实提升。生态环境改善任务艰巨。能源结构不够合理，主要污染物排放总量依然较大，生态建设长效机制尚不完善，弥补生态环境欠账还需付出艰苦努力。城乡统筹协调发展仍需大力推进。城乡区域发展和收入分配差距依然较大，规划建设与产业布局、生态保护之间的契合度不够高，城市现代化管理较为滞后。民生社会建设还有不少短板。巩固提升脱贫攻坚成果仍需加力，优质公共服务供给不足，群众在就业、社保、教育、医疗、养老、居住等方面还有不少难题。安全生产、网络安全、金融风险防控压力较大，社会治理面临许多新课题新挑战。政府自身建设有待进一步加强。行政效能和服务质量与市场主体和群众要求仍有差距，有的政府部门和机关干部工作标准不高，服务群众缺乏耐心，作风不实、担当不足、落实不力等问题依然不同程度存在，纠治“四风”顽疾还需持续用力。对这些问题，我们一定采取切实措施，认真加以解决。

二、2018年经济社会发展目标任务和工作重点

2018年是深入贯彻落实党的十九大精神的开局之年，是改革开放40周年，是决胜全面建成小康社会、实施“十三五”规划承上启下的关键一年。为切实做好今年及今后一个时期的工作，市委、市政府深入学习贯彻党的十九大精神和省委、省政府重大决策部署，在广泛深入调研论证、精准把握矛盾问题、认真听取意见建议的基础上，确定了“一个目标定位、四个着力建设、十个率先突破”的总体思路和工作布局。“一个目标定位”，就是以走在前列为目标定位，奋力实施新旧动能转换工程，奋力加快老工业城市全面振兴，奋力提升人民群众获得感幸福感安全感，努力使各项工作达到省内一流、全国先进。“四个着力建设”，就是突出新旧动能转换，着力建设新型工业化强市；突出文旅融合发展，着力建设文化名城；突出污染治理，着力建设生态淄博；突出统筹融合发展，着力建设现代化组群式大城市。“十个率先突破”，就是在重大项目建设、新型工业化强市建设、文化名城建设、生态淄博建设、现代化组群式大城市建设、现代服务业和开放型经济发展、人才工作和科技创新、全面深化改革、民生社会建设、全面从严治党等十个方面率先突破。同时，配套制定了具体的工作方案、实施方案和考核办法。

下步发展中，我们要深入学习贯彻党的十九大精神，以习近平新时代中国特色社会主义思想为指导，坚持稳中求进工作总基调，全面践行“五位一体”总体布局、“四个全面”战略布局和新发展理念，紧扣我国社会主要矛盾变化，按照高质量发展要求，对标佛山、无锡、宁波等先进城市，牢牢把握“一个目标定位、四个着力建设、十个率先突破”总体思路和工作布局，牢固树立政治导向、目标导向、问题导向、项目导向、业绩导向，以供给侧结构性改革为主线，以实施新旧动能转换工程为统领，统筹推进稳增长、促改革、调结构、惠民生、防风险各项工作，全力打好防范化解重大风险、精准脱贫、污染防治三大攻坚战，加快淄博老工业城市转型发展、全面振兴、走在前列。

全市经济社会发展预期目标建议为：地区生产总值增长7.5%左右，一般公共预算收入增长6.7%左右，固定资产投资增长8.5%左右，社会消费品零售总额增长10%左右，外贸进出口、实际使用外资平稳增长，城镇居民和农村居民人均可支配收入分别增长8%左右和8.5%左右，城镇登记失业率控制在4%以内，全面完成省下达的节能减排约束性目标。工作落实上，我们将坚持抓重点、补短板、强弱项，切实抓好六个方面：

（一）聚焦新旧动能转换，加力构建现代产业体系

以建设新型工业化强市为突破口，突出以“四新”促“四化”，扎实做好“高、新、轻、绿”四篇文章，着力构筑“753”现代产业体系，切实加快高质量发展步伐。

一是聚力推动工业迈向中高端。加快传统产业高端化。突出化工、传统机械、建材、冶金、纺织、轻工、陶瓷等七大传统产业，组织开展“技术改造突破年”活动，逐一明确规模以上企业转型升级方向和技术改造路径，集中抓好年度投资323亿元的306个重点技改项目，引导企业充分运用新技术、新材料、新工艺、新设备，对生产经营实行信息化智能化绿色化全程再造，推动企业自身及全产业链实现脱胎换骨式的提升。大力推进制造业与大数据、互联网、人工智能深度融合，高水平建设10家智慧工厂和100个智能车间，积极争创“中国制造2025”国家级示范区。加快动能培育新兴化。突出新材料、高端装备制造、生物医药、电子信息、新能源和节能环保等五大战略性新兴产业，实施新兴产业培育壮大行动计划，持续深化与大院大企的战略合作，集中抓好新能源汽车、新能源电池、铝基新材料、碳纤维新材料、电子芯片、MEMS传感器、机器人、高铁车轮、生物制药及装备、高效电机等领域的一批重点项目，着力培育10个新兴产业示范集群，战略性新兴产业产值占规模以上工业总产值比重提高5个百分点左右。加快发展生态绿色化。坚决摒弃资源依赖型发展模式，大力发展循环经济，推广节能减排技术和绿色节能产品。围绕深化供给侧结构性改革，切实加大“破”的力度，全面完成70万吨粗钢、60万吨生铁过剩产能化解任务，持续深化“散乱污”企业治理，坚定不移推进僵尸企业市场出清。

二是大力发展现代服务业。把发展现代服务业作为加快产业结构轻型化的关键举措，突出现代物流、文化旅游、现代金融等三大主导产业，集中抓好年度投资270亿元的163个服务业重点项目，服务业占比达到47%左右，现代服务业增加值占全市服务业比重达到50%左右。大力发展智慧物流，加快建设11个现代化物流园区，着力打造物流强市。抓好淄博新区商务中心金融街等金融聚集区建设，积极引进和发展银行、保险、证券、信托、基金、创投等现代金融业态，支持齐鲁股交中心、齐商银行、农商行做大做强，打造区域性金融中心。推进淄博经开区大数据小镇、上海斐讯临淄大数据中心、黄三角物联网产业园以及卓创资讯、隆众资讯等专业园区、骨干企业建设发展，推动信息服务业实现新突破。积极发展健康养老、教育培训、体育休闲、家政服务等生活性服务业，努力满足群众多样化多层次需求。

三是着力提升集约集聚发展水平。进一步优化产业布局。统筹抓好市五大园区和省级经济开发区建设，加快推进“张店、临淄、桓台、高新区”相邻园区，“桓台、高青”相邻园区，“淄川、博山”经济开发区，“张店南部、淄川北部、临淄西南部”区域等四个边界关联片区的互联互通、融合发展，集中打造产业聚集区、经济隆起带。强力推进化工产业安全生产转型升级，加快化工园区村庄搬迁，完成城市人口密集区危化品生产企业的搬迁、转产和关闭，推动化工产业加速迈向集约化、高端化、绿色化。进一步强化园区载体功能。各园区重点打造1-2个主导产业，集中力量壮大龙头企业、招引重大项目、完善产业链条，培育一批优势产业基地。加快建陶、耐火材料、日用玻璃等专业园区建设，规划建设电机泵类、智慧厨电、软体家具等10个以中小企业为主体的产业转型升级示范集群。鼓励社会资本参与园区标准厂房、仓储物流、融资租赁等服务平台建设，降低企业投资成本。进一步理顺园区体制机制。依法授予省级经济开发区县级行政审批权限，探索实行灵活高效的用人机制和分配机制，充分激发园区活力。

四是切实增强区域创新能力。以创建国家创新型城市为引领，深入实施人才强市和创新驱动战略，支持淄博高新区国家自主创新示范区建设，打造国内尖端水平、具有全球影响力的新材料研发中心和产业高地。积极推进中国钢研淄博研究院、中科院过程所绿色产业科技园等创新平台建设，深化与山东理工大学、淄博职业学院等校城融合发展，推动各类研发平台晋档升级。继续实施高新技术创新“双十”计划，强化产业技术创新联盟和重点企业创新主体作用，全市高新技术产业产值占比提高1个百分点以上。加强知识产权创造、保护和运用，组织实施创新创业“齐创行动”，加快建设IUIA（国际大学创新联盟）鲁中国际创客中心、桓台创智谷等优质平台，着力培育10个小微企业“双创”示范基地。全面落实市“人才新政23条”，组织实施人才队伍建设“六大工程”和“高技能人才十年培训计划”，努力让各类人才的创造活力竞相迸发、聪明才智充分涌流。

五是不断强化转型发展支撑。突出优质投资拉动。实施重大项目建设攻坚行动，全力抓好年度投资1030亿元的332个市重大项目，确保项目开工率和投资完成率达到100%。深化政银企合作，完善多元投资机制，提高普惠金融服务覆盖率，促进社会投资稳定增长。突出企业素质提升。精准扶持工业企业50强和创新型高成长企业50强跨越发展，支持驻淄央企省企加快重大生产力布局，鼓励上市企业通过并购重组等吸附优质要素，壮大骨干企业群体。推动中小微企业加快成长，省级中小企业“隐形冠军”达到20家以上。启动实施“10万企业家三年培训计划”，年内精准培训2万人以上，其中规模以上工业企业家2000人以上。注重激发和保护企业家精神，构建亲清新型政商关系。突出优势品牌培育。实施企业标准“领跑者”行动计划，深入推进企业管理对标，支持“老字号”创新发展，大力弘扬劳模精神和工匠精神，推动更多企业走“质量强企、品牌兴企”的内涵发展道路，夯实实体经济发展根基。

（二）聚焦污染防治攻坚，加力改善生态环境质量

自觉践行“绿水青山就是金山银山”理念，坚持系统谋划、精准施策、标本兼治，以铁的决心、铁的措施、铁的手腕抓严生态环境治理，推动生态淄博、美丽淄博建设迈上新台阶。

一是坚决打赢蓝天保卫战。实施全区域、全行业、全过程治理，紧紧抓住影响环境空气质量的主要矛盾，集中开展细颗粒物（PM2.5）和二氧化氮专项治理攻坚，巩固提升中央和部、省环保督察巡查成果，全年空气质量良好天数和“蓝繁”天数占比分别达到60%和75%。严格执行电力、钢铁、建材、水泥等重点行业排放限值，加快推进电力行业燃煤机组整合，禁止新上5万千瓦以下煤电机组。加强石化、有机化工、包装印刷、表面涂装、加油站等行业深度治理，开展用气企业达标排放专项整治，强化重型柴油车等移动污染源监管，切实减少挥发性有机物排放。大力深化建筑施工、道路交通、露天矿山、物流园区等重点领域扬尘污染防治，建筑工地全面落实“6个100%”环境管理要求。管住管好禁煤区，继续实施气代煤、电代煤改造，在其他区域推广民用清洁煤炭，全市煤炭消费总量控制在2830万吨以内。

二是持续深化水污染防治。扎实推进全流域综合治理，开工建设孝妇河博山段综合整治工程，完成淄川到马踏湖段全线整治和范阳河综合治理；实施淄河干流及其重要支流、乌河临淄—桓台段生态修复，加快构建“水润淄博”生态水系。封堵取缔河道排污口，搬迁改造光大水务一分厂等3座城镇污水处理厂，确保全市城镇污水处理厂排水全盐量达标。编制实施刘征水源地、大武地下水富集区生态保护和修复规划，关闭取缔非法自备井，全面消除黑臭水体和黑臭坑塘，所有河流稳定消除劣Ⅴ类水体，COD和氨氮浓度稳定达到Ⅳ类水体标准，集中式饮用水水源地水质达标率稳定在100%。

三是进一步加强生态保护和修复。严格落实生态红线和基本农田保护制度，深入推进博山区、沂源县国家重点生态功能区建设，加强原山和鲁山省级自然保护区综合管理，全面推行产业负面清单制度。深化矿山地质环境恢复治理，组织实施“3322”生态绿化工程，夯实城市绿色基底。加快化工园区危险废物处置中心建设和市医疗废物处置中心搬迁，扎实开展土壤污染综合治理，加大农业面源污染防治力度，确保生态环境安全。

四是切实完善生态建设长效机制。严格落实“党政同责、一岗双责”，加严项目环保审批，不断深化专项督查、提级调查和“双罚”制度，推动各级各部门更加自觉履行环保职责。全面落实河长制和环保网格化监管机制，建成纳污管网水质监控网络，实现空气质量自动监测镇（街道）全覆盖。加大“刑责治污”力度，持续深化宣传引导，推动绿色发展理念更加深入人心，让蓝天白云常驻淄博、绿水青山造福人民。

（三）聚焦文旅融合发展，加力推进文化名城建设

以塑造鲜明地域特色、鲜活城市形象、鲜亮文化品牌为主线，突出优势资源的深度挖掘、科学利用、有机整合，统筹推进文化产业和文化事业发展，努力增加优质文化产品供给，提升城市文化软实力。

一是加快齐文化传承创新示范区建设。切实抓好齐古城、齐国故城考古遗址公园、稷下学宫建设，管仲纪念馆和姜太公祠扩建等核心区重点工程，高水平举办齐文化节系列活动，不断增强齐文化影响力。加强陶琉文化、聊斋文化、鲁商文化、红色文化、孝文化、黄河文化、渔洋文化、丝绸文化、牛郎织女爱情文化等优质资源开发利用，努力促进创造性转化、创新性发展，共同讲好“淄博故事”。

二是深入推进文化与产业融合发展。组织实施文旅融合八大工程，突出抓好齐长城鲁山原山“三位一体”生态文化旅游带、华侨城文化旅游综合体、陶琉古镇、马踏湖旅游度假区、梦幻聊斋VR主题公园等一批重点项目，建设蹴鞠小镇、“国井”小镇、五阳湖休闲体育小镇等特色小镇，着力培育新亮点、新业态。注重高端品牌带动，推动齐王陵申报世界文化遗产，周村古商城和原山创建5A景区，高青县天鹅湖温泉慢城申报国际慢城，推进博山国家级风景名胜区建设，支持沂源县创建国家全域旅游示范区。加大市场开拓力度，积极发展智慧旅游，新建30处旅游公共服务中心，提升重点景区综合配套功能，让淄博成为更多游客了解和喜爱的旅游目的地。推进国家文化消费试点城市建设，加快发展创意设计、动漫游戏、影视制作、数字出版等文化业态，文化产业增加值占比达到4.8%。

三是着力提升城市文明水平。大力弘扬社会主义核心价值观，深化中国特色社会主义和中国梦宣传教育，建设焦裕禄干部教育学院；深入实施公民道德建设工程，推进群众性精神文明创建活动，巩固全国文明城市创建成果。健全现代公共文化服务体系，全面建成市文化中心，加快4个区县文化中心建设，完成7个区县档案馆新建工程，实现农村和城市社区文化中心全覆盖，让文化惠民活动越办越红火。进一步加强文物保护，继续实施“乡村记忆工程”，留住历史鲜明印迹，更好延展城市文脉。

（四）聚焦统筹融合发展，加力提升城市现代功能

按照“四位一体、组群统筹、全域融合”城市工作思路，高起点规划、高标准建设、高效能管理，着力塑造城市特色风貌，促进城乡一体化发展，加快建设现代化组群式大城市。

一是进一步做实“四组全”城市体系。以列入全国新一轮土地利用总体规划编制试点城市为契机，完善落实城乡规划体系，加快淄博新区、淄博经开区、文昌湖区等重点板块建设，建成高铁新城骨干路网及淄博北站、临淄北站；抓好北京路北延等8条城市骨干道路以及7座互通立交（高架桥）建设，加快形成“四位一体”骨干框架。加大组群统筹、全域融合力度，扎实推进G309青兰线改建等交通重点项目，积极争取上级支持，撤销G205、S294两座公路收费站。抓好济青高速、滨莱高速淄博段改扩建，开工建设沾临高速淄博段，争取济淄潍高速列入省建设规划。启动小清河复航淄博段建设。完成张东铁路电气化改造，加快张博铁路恢复客运前期工作并力争开工建设，抓好城市轨道交通规划报批并争取启动示范段建设，努力推动滨淄临高铁纳入国家规划。编制淄川—博山组团互联互通规划，策划实施一批促进两区协同发展的产业、文旅和基础设施项目，奠定与“四位一体”融合发展基础。扎实推进总投资23亿元的超高压等输变电工程。实施大数据发展战略，加强政务信息系统整合和资源共享，加快云计算中心、大数据共享交换平台、时空信息服务平台建设，着力打造新型智慧城市。

二是加快城市有机更新。深化全国“城市双修”试点，全力推进淄博火车站南广场片区建设改造，打造绿色智慧示范区。深入实施“三改三建”工程，抓好127个棚户区、90个老旧住宅小区改造；完成张店区、高新区所有背街小巷和其他区县城市建成区背街小巷整治改造，启动镇（街道）驻地背街小巷改造提升工程；新建改造张店区水产品批发市场等便民农贸市场18个、公共停车场148处、城市公厕161座，对城乡加油站公厕全面实行标准化改造。实施猪龙河柳泉路篷河段整治改造，抓好柳泉路（民祥路—淄河大道）、潘南路改造等一批市政路网工程，建设文昌湖—四宝山—马踏湖百公里绿道，进一步提升城市品质。深入推进城市管理标准化、智慧化、精细化、市场化和社会化，深化城市管理执法体制改革，完善网格化和数字化管理机制，集中开展城市社区、市容市貌、建筑垃圾、闲置烟囱、工业固体废弃物等专项整治，全面完成违法建设治理任务。进一步健全公共交通体系，加强城市网约车、共享单车规范管理。

三是大力实施乡村振兴战略。按照产业兴旺、生态宜居、乡风文明、治理有效、生活富裕的总要求，推动公共财政和社会资源更大力度向“三农”倾斜，将农业农村优先发展落到实处。深化农村土地制度和集体产权制度改革，壮大集体经济。坚持绿色兴农、质量兴农，切实保障粮食安全，大力发展生态特色农业、休闲观光农业、优质高效农业，支持社会资本参与农业开发，年内新增新型农业经营主体500家以上，带动农民持续增收。坚持以工补农、以城带乡，抓好国家新型城镇化综合试点，着力培育新生小城市、重点示范镇和特色小镇，用两年左右时间完成省级经济开发区规划区内村庄搬迁改造。深入开展新一轮乡村文明行动，推进美丽乡村标准化建设，抓好农村基础设施和公共服务提档升级，巩固提升城乡环卫一体化、饮水安全、农村“五化”、旱厕改造成果，三年内80%以上行政村达到美丽乡村建设标准。完善落实居住证制度，稳步推进镇改办、村改居，促进农业转移人口市民化，年内常住人口城镇化率提高1个百分点。

（五）聚焦增强区域活力，加力深化改革扩大开放

把改革开放作为推动转型发展的关键一招，努力破除各类体制机制障碍，努力提高更好利用两个市场、两种资源的能力和水平，为区域发展不断注入新动力、增添新活力、拓展新空间。

一是深化重点领域改革。持续深化投融资体制改革，加强政府引导基金实质性运作，加快地方融资平台转型发展；进一步扩大直接融资，制定推动企业上市五年计划，力争上市公司数量实现翻番。深化财税体制改革，扎实做好环境保护税和水资源税开征工作，抓好预决算公开、财政资金统筹整合、精准支持转型发展等重点环节，严控“三公”经费等一般性财政支出，提升资金使用效益和财政经济质量。进一步深化国有企业改革，健全企业法人治理结构，稳步推进国有资本整合重组；组建市国资委，将机关、事业单位兴办的各类实体逐步纳入监管范围，实现对经营性国有资产的统一监管。完善市、县（区）公共资源交易平台，推动各类公共资源交易应进必进。继续深化教育、医疗、物价、事业单位等重点领域改革。

二是以招商引资为突破口大力发展开放型经济。深入实施“四大招引工程”，抓住用好国家、省重大区域发展战略机遇，集中优质资源，调动各方力量，在引进实力雄厚的大企业以及大院大所上实现更大突破，推动一批支撑带动力强的重大产业项目以及新技术、新模式、新业态加快落地，全市投资亿元以上产业项目不少于125个，外来投资达到1150亿元。突出招引世界500强和行业领军企业，市有关部门牵头招引8家以上，各区县均实现新突破。推进外贸转型升级，深入开展“跨境电商进企业”专项行动，培育壮大周村区“绿方达”、高新区“齐贸通”等重点平台；大力发展服务贸易，支持博山区创建国家特色文化服务出口基地，年内全市货物贸易、服务贸易进出口总额分别突破700亿元和110亿元，品牌出口占全市出口份额提高到30%以上。积极参与“一带一路”建设，稳步推进国际并购和产能合作。全力争取淄博保税物流中心获批国家综合保税区，扩大“淄博号”中欧国际货运班列辐射半径，打造鲁中进出口货物集散中心。

三是进一步优化营商环境。深化放管服改革，最大限度削减行政审批事项、减少行政审批环节、减除无谓证明和繁琐手续、减轻企业税费成本压力，激发市场主体活力。着力完善政务服务大厅“一窗受理”机制，从进多个门办一件事变为进一个门办所有事。加快推行“互联网+政务服务”，统筹推进“多证合一”、证照分离、全程电子化登记，努力实现“不见面审批”；全面实现省市县乡村“五级联动”，政务服务事项全部在山东政务服务网公开办理。加快“信用淄博”建设，健全信息共享、部门联动的社会信用管理服务体系，落实守信激励和失信惩戒机制，努力营造诚实守信、公平竞争的市场环境。

（六）聚焦增进民生福祉，加力推进民生社会建设

大力践行以人民为中心的发展思想，切实解决群众最关心最直接最现实的利益问题，努力满足人民日益增长的美好生活需要，不断提升群众的获得感幸福感安全感。

一是巩固提升脱贫攻坚成果。坚持精准扶贫、精准脱贫基本方略，深入推进国家扶贫改革试验区建设，聚焦南部山区、太河库区、黄河滩区和特殊困难群体，特别是474个省扶贫工作重点村和100个薄弱村，多措并举，精准施策，确保建档立卡贫困人口稳定实现“两不愁、三保障”。加强贫困人口信息动态管理，健全扶贫政策体系，完善稳定脱贫工作机制，推动扶贫与扶志、扶智相结合，坚决做到真脱贫、脱真贫。

二是统筹抓好就业和社会保障。认真落实“零成本创业”等扶持政策，积极做好高校毕业生、农村转移人口、去产能分流职工等重点群体就业工作，着力解决结构性就业矛盾，全年新增城镇就业7.76万人以上。深入实施“全民参保登记计划”，进一步提高社会保障和服务水平，加强农村低保和特困群体救助工作，健全农村留守儿童和妇女、老年人关爱服务体系，努力做到应保尽保。全面落实中央、省住房制度改革要求，加强房地产市场运行监测管理，建立完善多元化住房保障体系。扎实推进医养结合，新建城市社区日间照料中心30家、农村幸福院100家。

三是提高教育、卫生等社会事业发展水平。优先发展教育事业，启动“国家义务教育优质均衡发展县（区）”创建，实施《2018-2020年消除大班额专项规划》，开工建设淄博新区高中、淄博十七中新校等学校，90%的中小学建成数字化校园；实施第三期学前教育行动计划，普惠性幼儿园占比达到80%以上。建成投用齐鲁医药学院新校区，支持山东工艺美院淄博陶瓷学院发展，提升高等教育、职业教育发展质量。深入推进“健康淄博”建设，建成投用市中心医院西院区、市中医医院东院区、市妇幼保健院新院区，启动市公共卫生中心建设；大力推行“互联网+医联体”和“第一村医”，完善提升镇、村（社区）医疗服务体系，继续抓好家庭医生签约服务，促进优质医疗资源上下贯通。进一步提高计划生育服务水平。创建全国基层中医药工作先进市。确保通过国家卫生城市复审。争创国家食品安全示范城市和全国农产品质量安全市，让群众吃得更加放心。丰富体育服务供给，推动体育事业与体育产业协调发展。

四是加强和创新社会治理。牢固树立底线思维和红线意识，深入推进法治淄博、平安淄博建设，实施城市社区综合治理两年行动计划，全面推行“四位一体”社区管理服务模式，城市社区网格员专职化率达到90%。建成四级综治中心，建设105个平安示范小区，深化视频监控全覆盖，构建立体化社会治安防控体系。一刻也不放松地抓严安全生产，全面完成重点行业规模以上企业安全风险管控体系和隐患排查治理体系建设，扎实开展“学规、懂规、践规”活动，大力强化专业化、网格化监管和“刑责治安”，不断深化危化品、道路交通、建筑施工、矿山、消防等重点领域专项治理，确保安全生产形势持续稳定。深入推进金融风险防控攻坚，加强重点担保圈综合治理，严厉打击逃废银行债务、非法集资、互联网金融诈骗等违法犯罪行为，坚决守住不发生区域性金融风险的底线。完善矛盾纠纷排查和多元化解机制，整合全市政务服务热线，畅通群众诉求表达渠道，维护社会大局和谐稳定。

三、努力建设人民满意的服务型政府

面对新时代新任务新要求，全市各级政府必须深入学习贯彻习近平新时代中国特色社会主义思想，以更加昂扬的姿态、更加过硬的本领、更加务实的作风、更加积极的作为，推动服务型政府建设不断取得新成绩。

旗帜鲜明讲政治。坚定执行党的政治路线，严格遵守政治纪律和政治规矩，坚决维护以习近平同志为核心的党中央权威和集中统一领导，在政治立场、政治方向、政治原则、政治道路上同党中央保持高度一致。牢固树立“四个意识”，坚定“四个自信”，认真贯彻执行中央和省、市委决策部署，确保政令畅通、令行禁止。扎实开展“不忘初心、牢记使命”主题教育，严格落实民主集中制各项制度，不断提高政治觉悟和政治能力。

深入推进依法行政。加强法治政府建设，自觉依照法定权限和法定程序履行职权，将政府活动全面纳入法治轨道。健全科学民主依法决策机制，完善重大行政决策法定程序及决策后评估制度，加强政府信息与政务公开，确保权力在阳光下运行。深化行政执法“三项制度”改革，切实做到严格规范公正文明执法。自觉接受人大法律监督、政协民主监督、社会和舆论监督，认真执行市人大及其常委会决议决定，主动听取民主党派、人民团体和社会各界意见建议，高质量办理人大代表建议和政协委员提案。

坚定不移从严治政。始终把人民利益摆在最高位置，牢固树立宗旨意识，认真倾听群众意见，真切了解群众需求，尽心竭力为群众办实事、解难题。严格落实中央八项规定和省、市委相关要求，驰而不息纠治形式主义、官僚主义等“四风”问题，推动作风建设向纵深发展。聚焦公共资金、国有资产、工程建设、民生保障等重点领域，创新完善监督管理机制，扎牢织密制度笼子。支持配合深化国家监察体制改革试点工作，加强审计监督，规范权力运行，坚持以零容忍的态度惩治腐败，切实营造风清气正的政治生态。

坚决践行实干担当。全面推进学习先进对标发展，大力弘扬求真务实、干事创业精神，深入一线查实情，立足实际谋实招，攻坚克难求实效。坚持一分部署、九分落实，推进政府工作项目化、具体化，明确时间表、路线图、责任人，紧盯不放抓全程，不达目标不罢手。深化完善“六个一”督察机制，大力破解“为官不为”“落实不力”问题，不断提高政府执行力和公信力。深入推进学习型政府建设，强化法治思维和专业素养，努力锤炼服务群众、推动发展的过硬本领，建设一支政治坚定、开拓创新、实干担当的高素质公务员队伍。

各位代表！新时代开启新征程，新使命展现新作为。让我们更加紧密地团结在以习近平同志为核心的党中央周围，在中共淄博市委的坚强领导下，不忘初心、牢记使命，团结实干、砥砺奋进，为全面建成小康社会，实现淄博老工业城市转型发展、全面振兴、走在前列而努力奋斗！
